# Adaptive Synaptogenesis Constructs Neural Codes That Benefit Discrimination

**DOI:** 10.1371/journal.pcbi.1004299

**Published:** 2015-07-15

**Authors:** Blake T. Thomas, Davis W. Blalock, William B. Levy

**Affiliations:** 1 Informed Simplifications, LLC., Earlysville, Virginia, United States of America; 2 Department of Computer Science, Massachusetts Institute of Technology, Cambridge, Massachusetts, United States of America; 3 Department of Neurosurgery, School of Medicine, University of Virginia, Charlottesville, Virginia, United States of America; Thayer School of Engineering at Dartmouth, UNITED KINGDOM

## Abstract

Intelligent organisms face a variety of tasks requiring the acquisition of expertise within a specific domain, including the ability to discriminate between a large number of similar patterns. From an energy-efficiency perspective, effective discrimination requires a prudent allocation of neural resources with more frequent patterns and their variants being represented with greater precision. In this work, we demonstrate a biologically plausible means of constructing a single-layer neural network that adaptively (i.e., without supervision) meets this criterion. Specifically, the adaptive algorithm includes synaptogenesis, synaptic shedding, and bi-directional synaptic weight modification to produce a network with outputs (i.e. neural codes) that represent input patterns proportional to the frequency of related patterns. In addition to pattern frequency, the correlational structure of the input environment also affects allocation of neural resources. The combined synaptic modification mechanisms provide an explanation of neuron allocation in the case of self-taught experts.

## Author Summary

One neural correlate of being an expert is more brain volume—and presumably more neurons and more synapses—devoted to processing the input patterns falling within one's field of expertise. As the number of neurons in the neocortex does not increase during the learning period that begins with novice abilities and ends with expert performance, neurons must be viewed as a scarce resource whose connections are adjusted to be more responsive to inputs within the field of expertise and less responsive to input patterns outside this field. To accomplish this enhanced, but localized improvement of representational capacity, the usual theory of associative synaptic modification is extended to include synaptogenesis (formation of new synapses) and synaptic shedding (rejection of synapses by a postsynaptic neuron) in a manner compatible with the original, associative synaptic modification algorithm. Using some mathematically simplifying assumptions, a theory is developed that predicts the algorithm's eventual outcome on expert neuronal coding, and then without the simplifying assumptions, computational simulations confirm the theory’s predictions in long, but finite periods of simulation-time (i.e., finite-sampling leads to stable connections, and thus, stable expert encodings).

## Introduction

Adaptive synaptogenesis [1–4] is designed to allocate neural resources in a thrifty manner or in a manner to regulate function. The three resources of concern are number of synapses, number of neurons, and firing-rate of the neurons. Inspired by the Bienenstock-Cooper-Munro (BCM) algorithm [5] and its forcing of a neuron to a predefined activity level, adaptive synaptogenesis achieves a similar goal that not only guarantees the average activity of a postsynaptic neuron but does so in a way that rations synapses.

**Fig 1:**
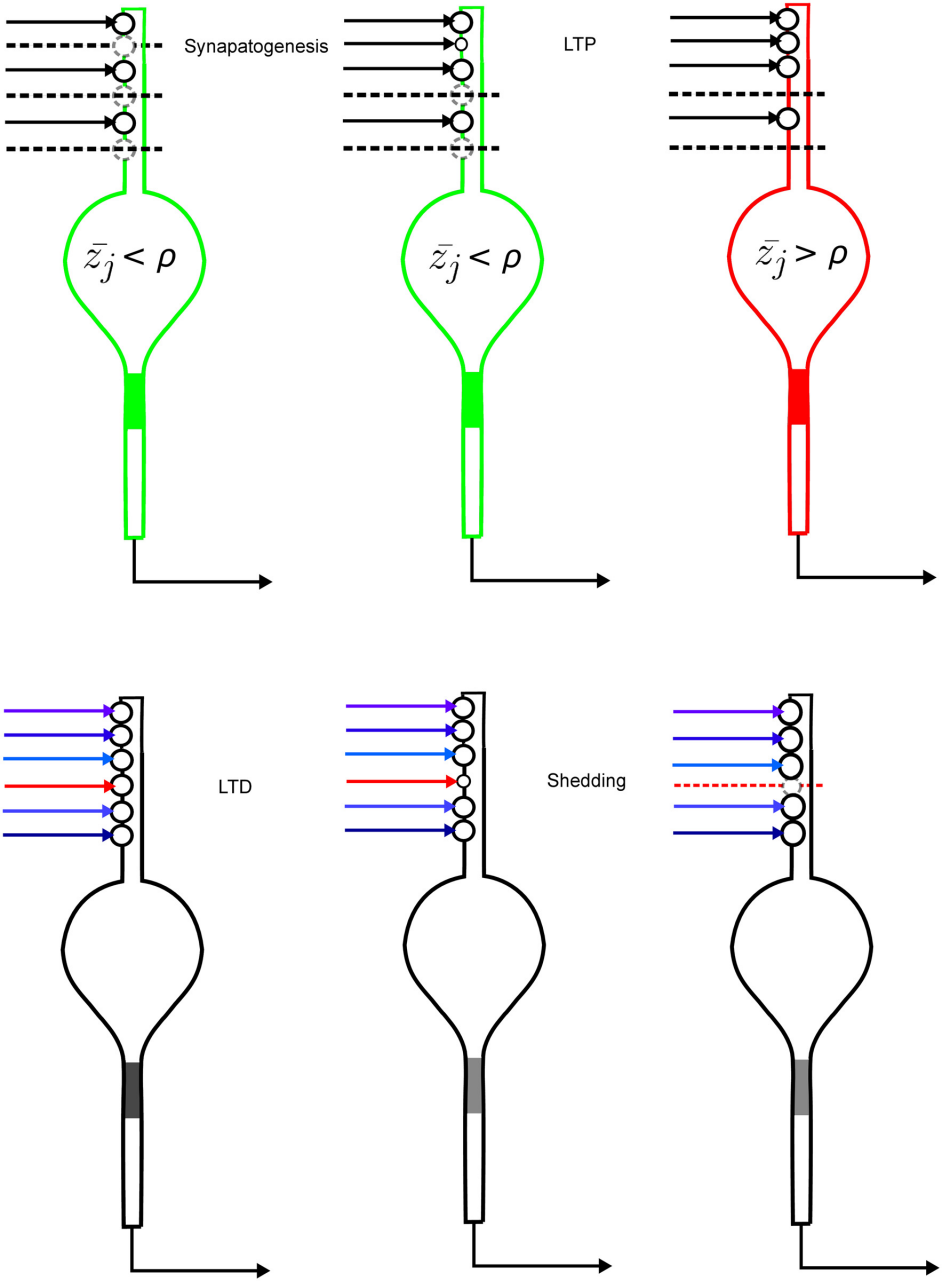
The three processes of adaptive synaptogenesis: Random synaptogenesis, bi-directional associative modification of existing synapses, and synaptic shedding. (Top) Synaptogenesis and positive associative modification (LTP). (Left) Receptivity for new innervation is below the cutoff firing-rate and three sites for a new synapse are indicated by the broken circles. (Center) Of the three locations, one new synapse is randomly generated (small complete circle), the uppermost available synapse. (Right) The new synapse increases strength due to the associative modification equation and positive correlation with enough of the other synapses on this neuron. Because of the increased excitation due to the new synapse, the post-synaptic neuron increases its average firing-rate above the prescribed design value; as a result of exceeding this value, receptivity for new innervation goes to zero (the broken circles disappear). (Bottom) Negative associative modification (LTD) and shedding. (Left) Having randomly acquired a certain set of inputs, one positively correlated subset (shown in shades of blue) dominates the excitation of the postsynaptic neuron while another input (shown in red) is negatively correlated with this subset. (Center) Associative synaptic modification decreases the weight of the uncorrelated input as indicated by the smaller circle. (Right) Associative synaptic modification further decreases the weight of the uncorrelated input, and because the weight value falls below the threshold, the synapse is shed.

Previously, adaptive synaptogenesis was used as a mechanism to produce compressive coding with small information losses [6–10]. It also successfully models developmental studies of ocular dominance [11–12]. Both results are achieved by postsynaptic neurons discovering implicit correlational structures within the input data space. Through the random acquisition and forced shedding of synapses, associated inputs find their way to the same neuron, and uncorrelated or anti-correlated inputs are ignored. As thus conceived, adaptive synaptogenesis consists of (1) a random Bernoulli process that selects a new excitatory connection between nearby axon *i* and postsynaptic neuron *j*; (2) once formed, associative synaptic modification controls the strength of each existing synapse, and this control includes the possibility of potentiation, depression, or no change of a synaptic weight; but with enough long-term depression, (3) shedding of a synapse occurs when the weight is appropriately weak (near zero for a sufficiently long time) (Fig 1). Critically, the possibility of forming a new synapse on neuron *j* is determined by *j*’s long-term average firing-rate.

Instead of compressive coding, the context for studying adaptive synaptogenesis here is self-taught discrimination. The motivating idea is that if one studies a particular field and its subject matter over a long enough period of time (perhaps the oft quoted ten thousand hours [13]) and if one studies over a wide enough variety of representative examples, the allocation of neurons in the cerebral cortex is enhanced for this particular concentrated field of study.

After a detailed description of the neural algorithm and the input data structures, we establish a mathematical theory that quantifies relationships (e.g. synaptic weights) required for stability (or lack thereof) of the neurons formed by this algorithm. Computational simulations follow these theoretical developments. The simulations demonstrate the development of stable neuron configurations without turning-off the algorithm. Moreover, these simulations also reveal the effect of input statistics—frequency of input patterns and the input correlational structure—on neuron allocation. As shown, the form of adaptive synaptogenesis used here produces neuron allocations that are appropriately biased by the statistics of the input environment (more experience produces more neurons devoted to the experience). Also revealed is an important effect of the input world’s statistical structure that can help or hinder this proportional neuron allocation.

## Methods

### Neurons

Here we study an adaptively constructed, feedforward network of McCulloch-Pitts neurons. The inputs are vectors with binary elements, *x*_*i*_(*k*)ϵ{0,1}, and the outputs are vectors with binary elements, *z*_*j*_(*k*). For the *j* ^*th*^ neuron, postsynaptic excitation is linear, *y*_*j*_(*t*) = Σ_*i*_ *x*_*i*_(*t*)·*c*_*ij*_(*t*)·*w*_*ij*_(*t*) with connection indicator *c*_*ij*_(*k*)ϵ{0,1}, with all weights *w*_*ij*_ positive, and output *z*_*j*_(*k*): = {1 if *y*_*j*_(*k*)≥*θ*, and 0 otherwise}. Threshold θ is 3.0 for dataset A and 0.8 for datasets B. The “sensory” input dimensions are 80 (dataset A) or 390 (datasets B) as described below. The number of postsynaptic neurons simulated is 2000 per dataset. Because there is no interaction between the outputs of these neurons (i.e. there is no feedback or lateral inhibition) and because there is no avidity rule [7], each neuron develops its connections independent of all other neurons.

Each neuron is initialized with one connection from a randomly chosen input line with weight 0.2.

### Adaptive synaptic modification

There are three distinct aspects of synaptic modification: synaptogenesis, associative synaptic modification, and synaptic shedding.

#### Synaptogenesis

Synaptogenesis, when it is allowed to occur, is a random Bernoulli process with parameter γ. Synaptogenesis onto postsynaptic neuron *j* changes *c*_*ij*_ from 0 to 1 when *u*_*ij*_(*τ*) = 1, where *u*_*ij*_(τ) is the generated Bernoulli random variable. Here, synaptogenesis only depends on the average output of *j* itself at time τ, i.e., ${\bar{\text{z}}}_{\text{j}}\left(\tau \right)$. This moving average is updated on each timestep: ${\bar{z}}_{j}(t)={\bar{z}}_{j}(t-1)\cdot (\alpha )+z_{j}(t-1)\cdot (1-\alpha )$. Moreover, to keep things as simple as possible and consistent with our requirement for asymptotic stability and certain observations of [14], *j*’s receptivity for new innervation, *r*_*j*_(τ), is either positive or zero; specifically, $r_{j}\left(\tau \right)=\left\{\gamma \text{ if }{\bar{\text{z}}}_{\text{j}}\left(\tau \right)<\rho ,\text{and 0 otherwise}\right\}$ where ρ = 0.09 for dataset A and 0.1 for datasets B1, B2, and B3. The parameter ρ is referred to as the minimum desired firing-rate because neurons that fire above rate ρ will no longer add new synapses.

When a new synapse is formed, its initial weight, *w*_*ij*_, is 0.2.

#### Associative synaptic modification

Associative synaptic modification is as suggested in [15]. With rate constant ε, *w*_*ij*_(*t*+1) = *w*_*ij*_(*t*)+*ε*·*c*_*ij*_(*t*)·(*x*_*i*_(*t*) – *E*[*x*_*i*_] – *w*_*ij*_(*t*))·*y*_*j*_(*t*), or equivalently,

$$\Delta w_{ij}(t):\;=w_{ij}(t+1)-w_{ij}(t)=\varepsilon \cdot c_{ij}(t)\cdot (x_{i}(t)y_{j}(t)-E[x_{i}]y_{j}(t)-w_{ij}(t)y_{j}(t))$$

where *t* advances one with each new input. Weights do not change if *c*_*ij*_(*t*) = 0 (i.e., the connection does not exist so there is no weight to change). Because the average firing-rate of all inputs *i* is positive (i.e. E[*X*_*i*_] > 0), an individual weight can be driven negative by the synaptic modification equation. However, such negative values of *w*_*ij*_ are prevented by the third mechanism controlling synapses and connectivity.

#### Synaptic shedding

A synapse is shed whenever its value goes below 0.01, formally *c*_*ij*_(*t*) = 0 if *w*_*ij*_(*t*) < 0.01. Thus no negative weights exist; moreover, new synapses, with their initial value of 0.2, are not immediately subject to shedding.

### Summary of basic definitions

A connection from input neuron *i* to output neuron *j* is indicated as *c*_*ij*_ (*t*)ϵ{0,1}. The strength (weight) of this connection is *w*_*ij*_(*t*)ϵ(0.01,1). Inputs are binary, i.e., *x*_*i*_ (*t*)ϵ{0,1}. Excitation of a neuron on a timestep is linear, *y*_*j*_(*t*) = Σ_*i*_ *c*_*ij*_(*t*)·*w*_*ij*_(*t*)·*x*_*i*_(*t*). There is no inhibition. A neuron, whose excitation reaches threshold, fires by the rule *z*_*j*_(*t*) = {1 if *y*_*j*_(*t*)≥*θ*, and 0 otherwise}. A weight is updated according to [15]; in particular, Δ*w*_*ij*_(*t*) = *ε*·*c*_*ij*_(*t*)·(*x*_*i*_(*t*) – *E*[*x*_*i*_] – *w*_*ij*_(*t*))·*y*_*j*_(*t*). On each timestep, a neuron’s moving-average of firing-rate, ${\bar{z}}_{j}$, is updated as ${\bar{z}}_{j}(t)={\bar{z}}_{j}(t-1)\cdot (\alpha )+z_{j}(t-1)\cdot (1-\alpha )$.

Synaptogenesis is controlled by ${\bar{z}}_{j}$ and a random variable: *u*_*ij*_ ϵ{0,1} where prob(*u*_*ij*_ = 1) = γ.

$$\begin{array}{l} \text{if( }c_{ij}(\tau )=0\text{ \& }{\bar{z}}_{j}(\tau )<\rho \text{ \& }u_{ij}(\tau )=1\text{ ),}\\ \text{ then }c_{ij}(\tau +1)=1\text{ \& }w_{ij}(\tau +1)=0.2,\\ \text{ else }c_{ij}(\tau +1)=\;c_{ij}(\tau ). \end{array}$$

A connection/weight is shed whenever it falls below 0.01; that is,

$$\text{if ( }w_{ij}(\tau )<0.01\text{ ), then }c_{ij}(\tau +1)=0\text{ \& }w_{ij}(\tau +1)=\emptyset .$$

### Parameters

There are two sets of parameterizations. There is one parameterization for dataset A and one parameterization for datasets B1, B2, and B3. However, many parameterizations were examined for each dataset, and in fact there are ranges of parameter settings for which the generic results presented below are valid. In this case ‘valid’ means a parameter set that produces stable connections and postsynaptic neurons that exceed the desired minimum firing-rate. For the results presented here, the parameterizations produce a relatively large number of synapses per neuron compared to other valid settings. The parameterizations are listed in Table 1. Neuron parameterizations that change between dataset A and datasets B1, B2, and B3 are threshold to fire (3.0 versus 0.8) and minimum desired firing-rate (9% versus 10%).

**Table 1:** Network parameters.

	Dataset A	Datasets B
***ε***	0.001	0.001
***α***	0.95	0.99
***γ***	0.001	0.001
**initial connections**	1	1
**initial synaptic weight**	0.2	0.2
**threshold for shedding**	0.01	0.01
***ρ***	0.09	0.1
***θ***	3.0	0.8

### Time-scales

As an explicit part of the model, there are three time-scales: i) the shortest is the neuron update (fire or not, given an input); ii) the next in duration is synaptic modification of existing synapses, which occurs every timestep; and iii) the slowest time-scale, which occurs after each training block, synaptogensis and shedding. In one cycle, all input vectors are presented to the network. A training block of inputs equals 10 cycles (e.g. if there are 50 input states, a block occurs after the network is presented with 500 inputs). The length of the simulations are shown in Table 2.

**Table 2:** Simulation parameters.

**block length in cycles**	10
**number of blocks presented**	2000
**total cycles**	20000

For each postsynaptic neuron the input blocks are repeated until no synapses are gained or lost for 200 blocks. At this time, a neuron’s synapses are assumed stable. At this point, as shown in the results, the synaptic weights have achieved their predicted values.

### Network analysis

S1 Code contains the Matlab program used for simulations. There are 2000 neurons simulated per dataset; this large number serves the purpose of producing accurate statistics. However, because the synaptic modification algorithms used here yield feed-forward networks with excellent data compression and little information loss, certain analyses only make sense when the number of neurons are much fewer than 2000; in particular we limit the number of randomly sampled neurons to 1 through 50 out of the 2000.

#### Statistical dependence

Statistical dependence is a measure of redundancy. A decrease in statistical dependence is called ‘compression’. Statistical dependence is calculated by summing the Shannon entropy of each dimension (input line) and then subtracting the Shannon entropy of the entire input set. It is only evaluated for dataset B1.

#### Discrimination errors

The output of the network is decoded using a supervised centroid rule. First, the centroid of the output vectors of each category is calculated from the training set. Then, each output vector (from the training and testing set) is categorized by selecting the closest centroid (Euclidean distance). If this selected centroid is from the appropriate category, there is no error; otherwise there is an error.

#### Neuron allocation index

The 2000, adaptively formed neurons are evaluated with a novel input set (a testing set). The primary evaluation is the neuronal allocation index (per category), i.e. the fraction of total firings of the 2000 neurons produced by the inputs drawn from a single category. For example, suppose that ten neurons fire a total of 100 times to an input set with three categories: category one accounts for 18 of these firings, category two accounts for 32 of these firings, and category three accounts for the remaining 50 firings. Then, category one, two, and three will have neuron allocations of 0.18, 0.32, and 0.5, respectively.

### Datasets

**Fig 2:**
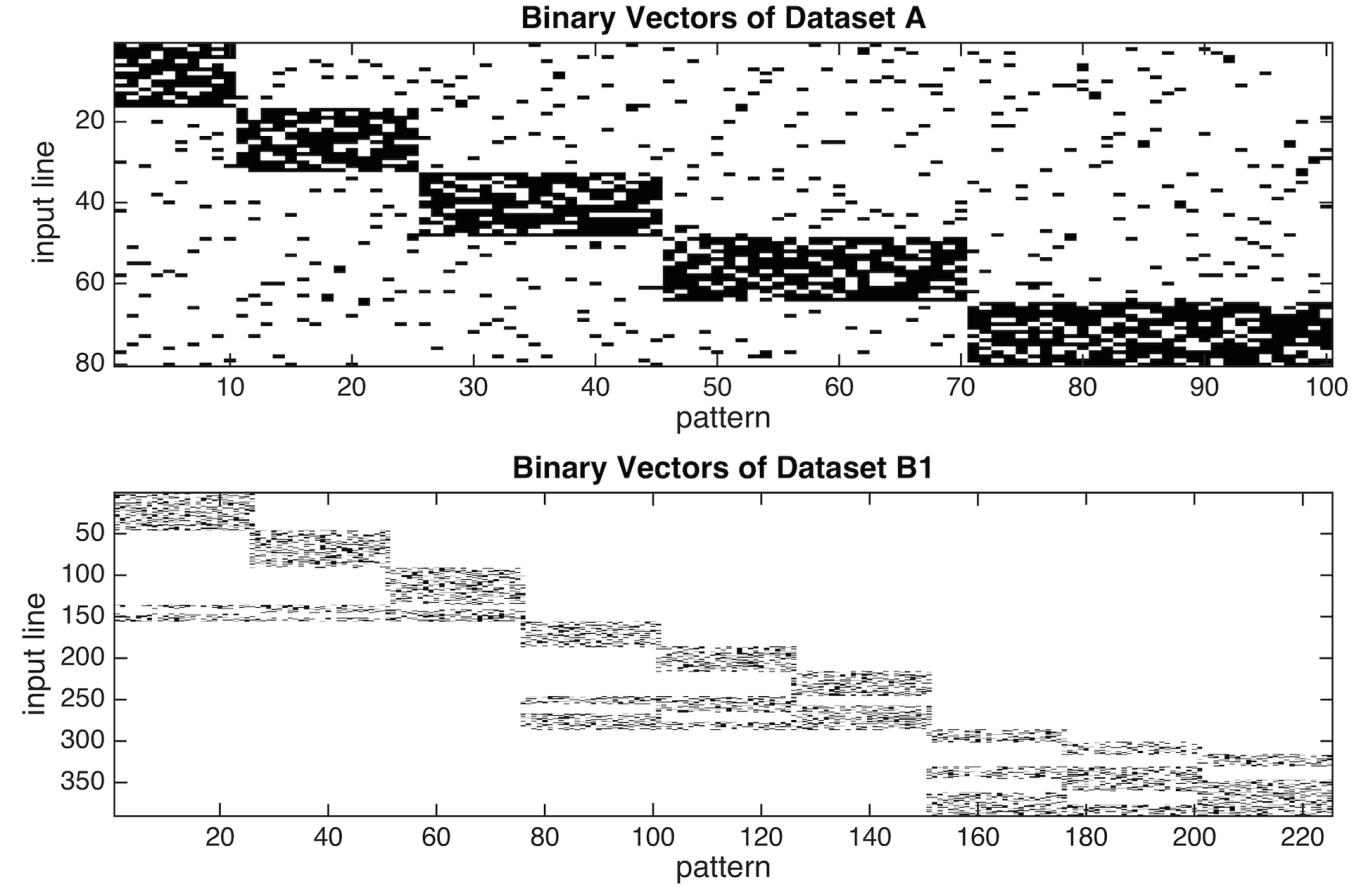
Randomly generated input vectors. (A) shows the 100 patterns of dataset A. An input pattern is built by perturbing one of five binary, 80-dimensional orthogonal vectors, see methods. Each of these five orthogonal vectors can be considered the central, unseen prototype pattern that defines the five distinct categories. The perturbation rule randomly complements two 1's and two 0's of such a prototype. Black pixels represent 1’s, and white pixels represents 0’s. Note the different relative frequencies of the categories: 10, 15, 20, 25, and 30%. (B) shows the 225 patterns of dataset B1. A pattern is built by perturbing each one of nine binary 390-dimensional vectors, see methods. Each of these nine vectors can be considered the central, unseen prototype that defines a distinct category. The perturbation rule randomly selects 20 out of 60 input lines from the central pattern to be active. Black pixels represent 1’s and white pixels represents 0’s. Note the three orthogonal super-categories, and note the differing overlaps of the categories across super-categories. The equal frequencies of each category only applies to dataset B1.

Neuron construction is driven by repeated presentation of patterns from a predetermined dataset. Four different input environments are studied (Table 3). The first dataset (dataset A, see Fig 2A and S1 Dataset) has 80 input lines. There are five orthogonal prototypes that define the corresponding categories: the five prototypes correspond to a higher probability of firing within one of five distinct sets of input lines 1–16, 17–32, 33–48, 49–64, or 65–80. The five exemplars are generated from these prototypes, and presented with relative frequency 0.1, 0.15, 0.2, 0.25, and 0.3 for a total of 100 input patterns.

**Table 3:** Datasets.

	Dataset A	Dataset B1, B2, B3
**input dimensions**	80	390
**axons active per pattern**	16	20
**number of input categories**	5	9
**positive noise (0 to 1)**	Yes	No
**negative noise (1 to 0)**	Yes	Yes

In the case of dataset A, each prototype is randomly perturbed such that the total number of active input-lines for each generated pattern remains constant. Specifically, two randomly selected active input-lines of the prototype are inactivated, and two randomly selected inactive input-lines of the prototype are activated. Fig 2A illustrates the binary input vectors of dataset A. Note the small amount of overlap between the input patterns only occurs due to noise.

Dataset B1 is much more complex in terms of relationships between input vectors. This set consists of nine categories each with its own prototype. Each of the nine individual categories has the same relative frequency (11.1%). The nine categories can be partitioned evenly into three super-categories: I, II, and III. Fig 2B visualizes the input set. These inputs are coded as 390-dimensional binary vectors. Between super-categories, the input patterns are orthogonal. Within a super-category, the prototypes and the patterns they generate overlap; the degree of overlap varies as a function of the super-category. Within the three super-categories overlap increases from 5 to 10, and then 15 input lines for super-categories I, II, and III, respectively. Each super-category has some input lines that are activated by only a single category; other input lines of this super-category are shared by two of the three categories; and the remaining input lines of the super-category are shared by all three categories. Super-category I has the least overlap: category A has 45 potentially active input lines that belong only to category A, 5 that belong to A and B, 5 that belong to A and C, and 5 that belong to A, B, and C for a total of 60 potentially active input lines per category. Super-category II has more overlap between its three categories (D, E, and F). There are 30 potentially active input lines that belong only to category D, 10 that belong to D and E, 10 that belong to D and F, and 10 that belong to D, E and F for a total of 60 potentially active input lines. Super-category III has the most overlap. There are 15 potentially active input lines that belong only to category G, 15 that belong to G and H, 15 that belong to G and J, and 15 that belong to G, H, and J for a total of 60 potentially active input lines. Thus, each category has a total of 60 potentially active input lines.

Exactly 20 of the 60 potentially active input lines from each category are pseudo-randomly chosen to be active for each pattern. None of the super-category’s designated inactive input lines are turned into active input lines.

**Fig 3:**
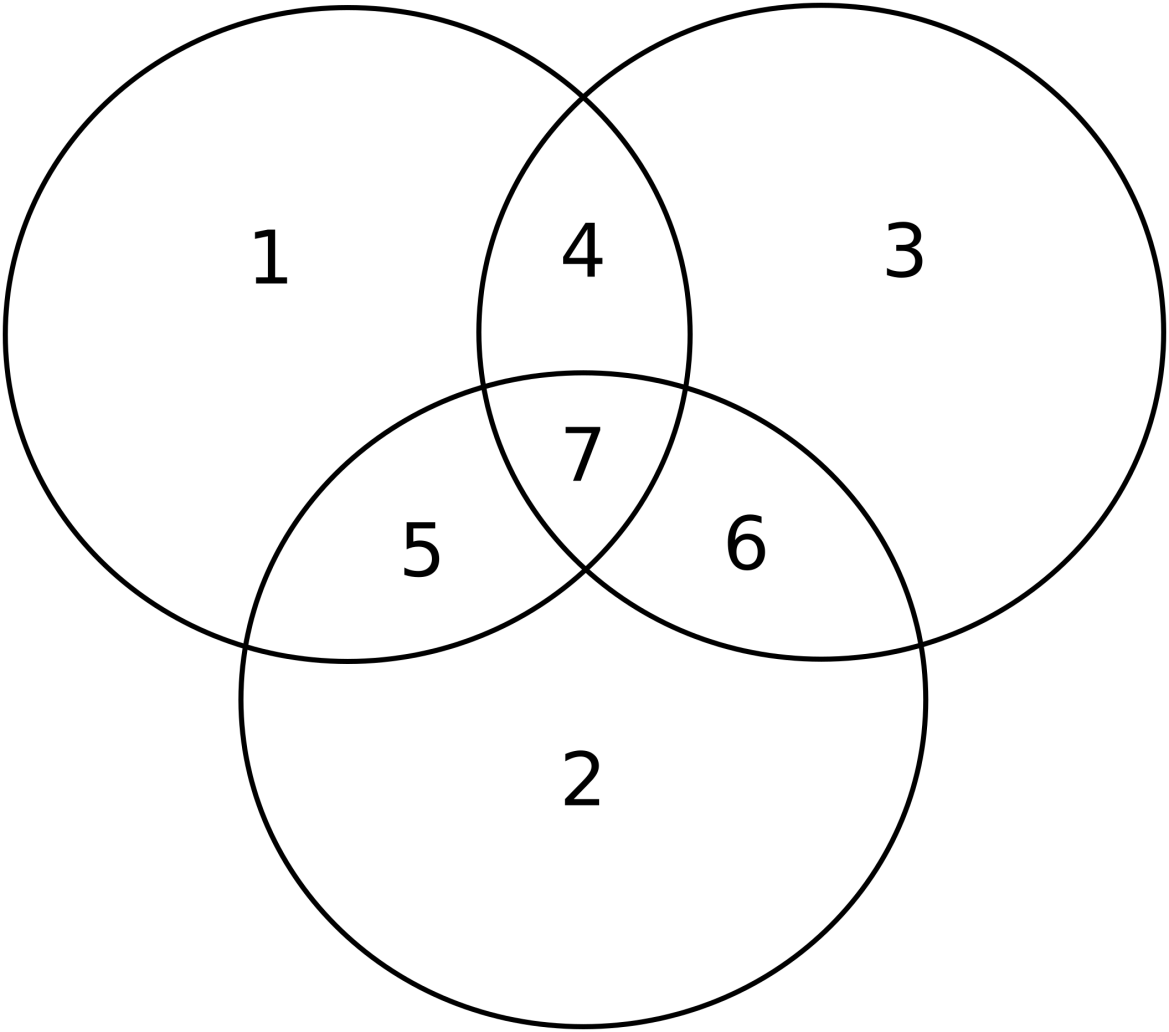
A visualization of super-category II of the B datasets. All three super-categories have seven constructed regions of overlap, this visualization shows the proportional overlap for the categories D, E, and F that make up super-category II. Category D is a union of the possibly active input lines from sub-regions 1, 4, 5, and 7. Similarly, Category E is a union of the possibly active input lines from sub-regions 2, 5, 6, and 7. Lastly, Category F is a union of sub-regions 3, 4, 6, and 7. Note that 30 input lines define sub-region 1, 10 define sub-region 4, which is shared between D and F, 10 define sub-region 5 which is shared between D and E, and 10 define sub-region 7 which is shared by D, E, and F.

Datasets B1, B2, and B3 are carefully constructed to illustrate the effects of category overlap (Fig 3 and S1 Dataset) versus category probability on neuron allocation. Datasets B2 and B3 are constructed in a similar way as dataset B1, but with different relative frequencies for each category. Dataset B2 has relative frequencies: 0.13, 0.13, 0.13, 0.11, 0.11, 0.11, 0.098, 0.093, and 0.093. Dataset B3 has relative frequencies: 0.18, 0.17, 0.15, 0.12, 0.11, 0.087, 0.063, 0.058, and 0.053.

## Results

### Theory

The most important idea of this section is that there is a mathematical derivation that characterizes the stable connectivities for a feedforward neuron whose connections are governed by adaptive synaptogenesis.

This theory’s convergence results provide a means for identifying stable configurations when simulations are performed. Going in the other direction, and of secondary importance is establishing the relevance of the theory via simulations because the theory requires multiple infinities of sampling: thus, simulations must be used to establish the existence of parameterizations capable of achieving the predicted, stable connectivities.

The stable weight values on a neuron with a stable connectivity are a function of the subspace covariance matrix that arises from the set of input lines received by this neuron. For example, one of our input environments is a random vector of 390 distinct input lines (axons arising from distinct neurons which may or may not be correlated in activity). Out of these 390 lines, a postsynaptic neuron may acquire a small fraction of this number, for example 20 input lines. Such an acquired set defines a subspace of the original space, and just as there is a 390-by-390 covariance matrix associated with the full input space, there is a specific 20-by-20 covariance matrix associated with the subspace defined by this neuron's input connections. Then for this neuron (call it *j*), we can also associate a dominant eigenvector and its associated eigenvalue arising from *j*'s covariance matrix. A simple theorem states that the weights of these inputs are proportional to this subspace’s dominant eigenvector. (A pleasing result since this vector maximizes the information throughput compared to all other linear, *n*-by-*1* input filters for a given *y*.) Moreover, the theorem below tells us 1) the proportionality constant that scales this eigenvector to the stable weight values, 2) the average excitation of *j*, and 3) the variance of *j*'s excitation.

In what follows, we assume that ε is a small positive constant and assume synaptic modification has been going on for a long time with a fixed set of input lines. Therefore the synaptic weights, i.e. the column vector *w*(*t*) for neuron *j*, change very slowly and thus can be treated deterministically. For a fixed set of input lines, each input-activation is random column vector *X*(*t*) (with realizations *x*(*t*)) with mean value E[*X*]. Via the definition of excitation, *y*(*t*) = *x*(*t*)^*T*^ *w*(*t*), the average excitation is E[*Y*] = E[*X* ^*T*^ *w*]. As noted above, the weights can be treated as a constant; thus in the limit, the mean excitation is E[*Y*] = E[*X* ^*T*^]*w*(∞). The variance of this excitation arises from the covariance matrix of the input to this neuron *j*. That is, define *j*’s covariance matrix of its input space as *Cov*(*X*): = E[*XX* ^*T*^] − E[*X*]E[*X* ^*T*^], and then note that

$$w{\left(t\right)}^{T}Cov(X)w(t)=E[w{\left(t\right)}^{T}XX^{T}w(t)]-E[w{\left(t\right)}^{T}X]\;E[X^{T}w(t)]\;=E[Y^{2}]-E{\left[Y\right]}^{2}=Var(Y)$$ (1)

Finally, define λ_1_ to be the largest eigenvalue of this covariance matrix and *e*_1_ as its associated eigenvector of unit length (the so-called dominant eigenvector).

**Theorem**. Assuming a stable set of input weights is achieved via the synaptic modification equation Δ*w*_*ij*_ = *ε*(*X*(*t*) – *E*[*X*] – *w*(*t*))*X*(*t*)^*T*^ *w*(*t*) operating along with the shedding rule then,

$$\begin{array}{l} E\left[Y\right]=\lambda_{1}\\ w(\infty ):\;=\underset{t\to \infty }{\text{lim}}w(t)=ke_{1},\text{ where}\\ k=\sqrt{Var(Y)/E[Y]}. \end{array}$$ (2)

Proof.

By definition, stability implies $\underset{t\to \infty }{\text{lim}}E[\Delta w(t)]=0$. Then, taking this same expectation and limit on the other side of the synaptic modification equation yields E[Δ*w*(∞)] = 0 = ε((E[*XX* ^*T*^] − E[*X*]E[*X* ^*T*^]) *w*(∞)* – w*(∞)E[*Y*]), or ε(*Cov*(*X*) *w*(∞) – *w*(∞)E[*Y*]) = 0, which implies

Cov(X)w(∞) =w(∞)E[Y]. (3)

Note that (3) is the eigen-equation, and the shedding rule guarantees all weights are positive while the synaptic modification equation guarantees *w*_*ij*_ is bounded from above because *X*–E[*X*] < 1. Therefore because *y* is bounded both below and above, convergence is implied. With our old synaptic modification rule based on a correlation matrix of a non-negative input, the Perron-Frobenius (PF) theorem implies that the dominant eigenvector (associated with λ_1_) is in the all-positive orthant. Here however, without an all-positive covariance matrix, we must conjecture an extension to PF (see below). Thus, by this perturbation conjecture, the synaptic weights align with *e*_1_ (proving 2). Now designate an unknown positive constant *k* and define *w*(∞) = *ke*_1_. Pre-multiplying (3) by $e_{1}^{T}$ quickly yields (2): $e_{1}^{T}Cov(X)w(\infty )=e_{1}^{T}w(\infty )E[Y]$ implying $\lambda_{1}^{}e_{1}^{T}ke_{1}^{}=e_{1}^{T}ke_{1}^{}E[Y]$, producing the result E[Y] = λ_1_.

For (2), pre-multiple both sides of (3) by *w*(∞)^*T*^, and note that by Eq (1) the left hand side is *var*(*Y*) while the right hand side yields *k* ^2^E[*Y*]. Thus,

$$k=\sqrt{Var(Y)/E[Y]}$$

If a neuron happens to acquire enough synapses, a valid central limit theorem (with mean and variance of the excitation values) would even tell us where threshold should be placed to produce the desired activity level. That is, the right-hand tail, beginning at threshold, yields the fraction of times a neuron fires to a randomly sampled input.

This theorem assumes convergence of all algorithmic processes. However there is an important exception to the convergence hypothesis. Certain input configurations will never produce stable connectivities nor achieve their algorithmically guaranteed firing-rates. Sensibly, such neurons might be killed-off; such neurons might lower their firing threshold; or from another perspective, such an input configuration will be very unlikely to exist. For example, we must conjure an input environment in which a set of input patterns is orthogonal to all others (thus very unlikely) and the probability of a member of this set occurring is less than the receptivity cutoff. For example, suppose synaptogenesis remains positive until a neuron fires 10% of the time. Suppose a subset of patterns occurs 9% of the time and that this set of patterns is orthogonal to all the other patterns. If a subspace of this set with its positively correlated input lines gains a controlling influence on a postsynaptic neuron, then any other input line not positively correlated but acquired through synaptogenesis will have its weight decreased by the synaptic modification equation and then it will eventually be shed. Nevertheless, no matter how many positively correlated input lines are acquired, synaptogenesis continues never to halt (because postsynaptic firing will converge to 9%, a value below the required 10% that halts synaptogenesis on such a neuron).

#### Extending the Perron-Frobenius theorem

For any one neuron, the algorithmic construction of its all-positive weight vector hinges, in a mathematical sense, on a perturbation of the classic PF result. The classic PF result specifies a sufficient condition for an all-positive dominant eigenvector, specifically an all-positive (and positive definite) symmetric matrix. However, this sufficient condition is not a necessary condition. In fact, one can mildly perturb an all-positive covariance matrix into a matrix with a relatively small number of not-too-negative entries, and it is still the case that the dominant eigenvector is all-positive. Although we know of no theorem that makes, much less quantifies this statement, it is easy to construct such matrices using Mathematica. Moreover, by inspection, we know that the subspaces of the covariances associated with our data sets and the simulation-discovered input vectors have such negative entries while at the same time have an all-positive dominant eigenvector.

From a purely algorithmic perspective, it is the shedding rule that enforces the all-positivity of a neuron’s inputs; thus it is the shedding rule that allows a neuron’s input covariance matrix to be such a suitably mild perturbation of a positive matrix. An issue that needs to be resolved from the combined perspective of random matrix theory and neuroscience is the relative abundance of such mildly perturbed covariance matrices. In regard to this combined perspective, it seems that one needs to examine distributions of covariance entries. For example, here is a sketched proof-heuristic for such a perturbed PF result with an explicit distributional assumption on covariance entries.

There are two steps to the heuristic. First note that the algorithm being used to find the dominant eigenvector can be replaced by the von Mises power method. Then, if for any symmetric matrix *M*, *M* raised to some integer power *p*>1 is all-positive, then the PF result can be applied even if *M* itself is not all-positive. Here we just consider *p* = 2 and the construction of random vectors that are mostly positive but whose inner products with each other are, with high probability, positive. Construct two random vectors each with *n* elements. Suppose the random elements are chosen from a uniform distribution that ranges from—a to 2a (a>0). Then, when *n* is large, 1/3 of the values fall into one of the three ranges. [-a,0), [0,a), and [a,2a]. Now it should be easy to see that the inner product of two such high-dimensional vectors tends to be positive (and the greater the positive offset of the mean from zero the faster this occurs). That is, consider the partitioned inner products of the elements in the range of [a,2a] times those in the other two ranges [-a,0) and [0,a). For large *n* these two inner products will sum to nearly zero; the inner product of values in [-a,0) times (0,a) will be negative, but this sum approximately matches the positively valued inner product of the [-a,0) elements with the other vector’s (-a,0) elements, and then an even greater tendency toward positivity occurs when we add in the positive inner products produced by the two matched partitions of the non-negative elements; thus, the grand total inner product is greater than zero with high probability. Finally, note that it is such inner products that populate *M* ^2^ if *M* is randomly constructed with such a distribution of covariances.

### Simulations

#### Convergence to stable weights

The results of this paper and their interpretation hinge on the stability of the adaptively formed neurons and their input synapses. Because the underlying theory assumes that time goes to infinity multiple times, there is reason to question the physiological applicability of the theory. Here simulations show that, for appropriate settings, the three dynamic aspects of synaptic modification can be combined to produce stable connectivity and stable weights; that is, synaptogenesis and shedding eventually come to a halt and the synaptic weight vectors (both in terms of direction and length) converge to the values predicted by the theory.

**Fig 4:**
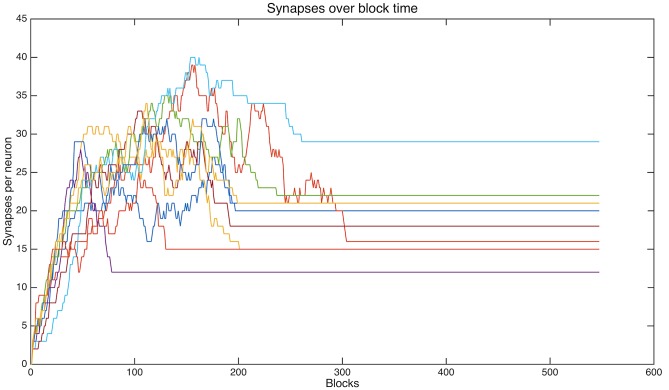
Driven by the adaptive algorithm, synapses are acquired and discarded but eventually a stable connectivity is achieved. This illustration follows the total connections for each of 10 representative neurons in one simulation as a function of number of blocks of input presentations. Note the wide distribution, across neurons, of the time-to-stable connectivity. Nevertheless all neurons illustrated here achieve stable connectivity by block 310 (the upper orange-red line), but one neuron achieves a stable connectivity as early as block 82 (purple).

In all of the simulations that follow, the synaptic weights are stable and connections are no longer being added or shed. Fig 4 shows the number of synapses for ten representative neurons as a function of the number of input blocks. For the last 200 blocks, there is no change in the number of synapses on each neuron. Of course these data could arise from a tread-milling effect in which each new synapse generated is exactly matched by a synapse shed, but the next evaluation denies this possibility. Therefore synaptogenesis and shedding have ceased.

Just as predicted by (2) of the theorem, each neuron has final weights proportional to the dominant eigenvector of its local covariance matrix (i.e. the subspace defined by the neuron’s input lines). For any neuron *j* the constant of this proportionality is equal to the square root of the variance of its excitation value, *y*, divided by the mean of its excitation value as described by Eq (2). For example, the dominant eigenvector of the local covariance matrix of neuron 28 from the simulations with dataset B1 is [0.192, 0.107, 0.305, 0.226, 0.307, 0.123, 0.416, 0.394, 0.608], and the weights for that neuron are [0.169, 0.094, 0.269, 0.198, 0.27, 0.109, 0.366, 0.347, 0.535]. The element-by-element ratio of the eigenvector to the weight vector is 0.880 for all elements (variance is 10^−27^). In fact, the square root of the variance of *y*_*j*_ divided by E[*Y*_*j*_] (*k* from the theorem) is 0.882 (a difference of less than 0.23%). Thus, the simulations confirm the theory, even though the theory requires an infinite number of training blocks.

#### Convergence and dynamics

Although convergence occurs for all datasets studied here, the time to converge to stable weights is not uniform across input categories. For dataset B1, the neurons that are captured by super-category I take significantly more training blocks to converge than neurons captured by super-category III (t-test, p = 9.15 * 10^−61^, Table 4). Thus, time to convergence for neurons learning about one particular super-category is negatively correlated with the number of neurons captured by this super-category (see neuron allocation below). Also, neurons that are captured by super-category I have significantly more synapses than neurons captured by super-category III. Thus, time to convergence for a super-category is positively correlated with the mean number of synapses on neurons captured by that super-category.

**Table 4:** Synapses and convergence.

super-category:	I	II	III
**mean synapses per neuron**:	22.8	22.2	16.6
**mean blocks to convergence**:	280.2	212.6	178.7

#### Characterization of the recodings

Feed-forward networks formed from such adaptively generated neurons have been characterized as efficient means of compressing patterns while preserving most of pattern information [6, 8–9]. Although compression is not the issue here, it still can be quantified, and indeed, compression occurs. However, as is the case with the antagonism between compression and mutual information in the earlier studies, there is an inverse relationship between pattern recognition errors and statistical dependence when the independent variable is number of post-synaptic neurons.

**Fig 5:**
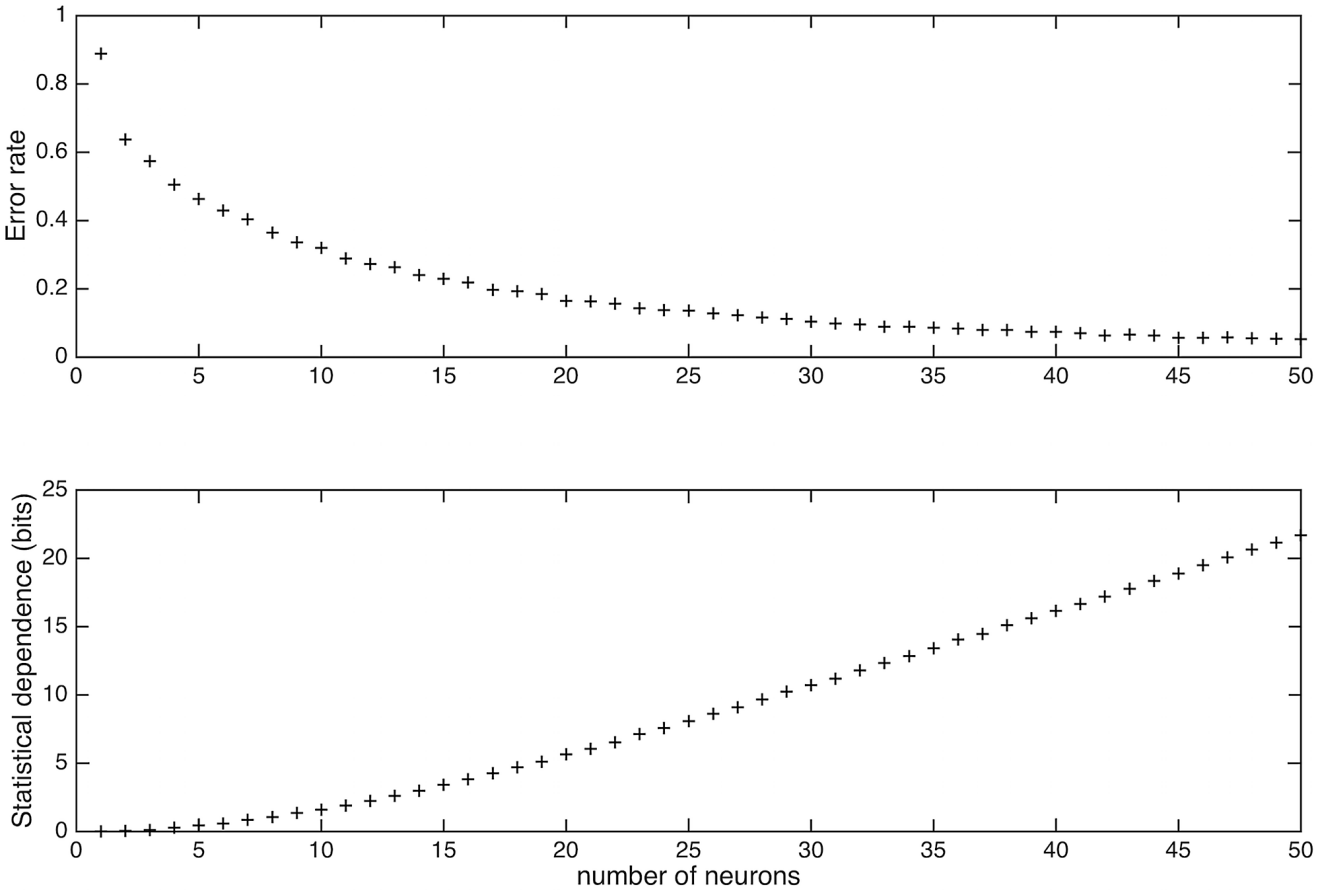
Error rate and statistical dependence depend on network size (Dataset B1). (A) As the number of postsynaptic neurons in a network increases, decoding error-rate monotonically decreases. A 10% error rate is reached once there are 34 neurons, and the amount of error continues to decline reaching 5.2% error at 50 neurons. (B) As the number of neurons increase, statistical dependence monotonically increases. When 34 neurons are sampled, statistical dependence is 12.84. Note that this is much less than the input statistical dependence of 102 bits.

As indicated in Fig 5, the discrimination error rate of networks with 10 neurons is quite high (32%) while statistical dependence is extremely low (1.61 bits). But for networks defined by 30 randomly sampled neurons, the mean error rate decreases to 10.42% while mean statistical dependence increases 10.72 bits. For networks of 50 randomly chosen neurons (out of 2,000), the mean error rate decreases to 5.2% and mean statistical dependence increases to 21.68 bits (still a substantial reduction from the input statistical dependence 102.4 bits).

Since these feed-forward networks are reducing statistical dependence while achieving a reasonably low error-rate for unsupervised neurons, we conclude that the algorithm produces efficient data compression.

#### Neuron allocation

Recall that the basic conjecture: the more frequent the exemplars of a category, then the greater the neural representation for that category [10]. Thus, the fraction of neurons devoted to the different categories of an input space is the measurement that produces our most important result.

**Fig 6:**
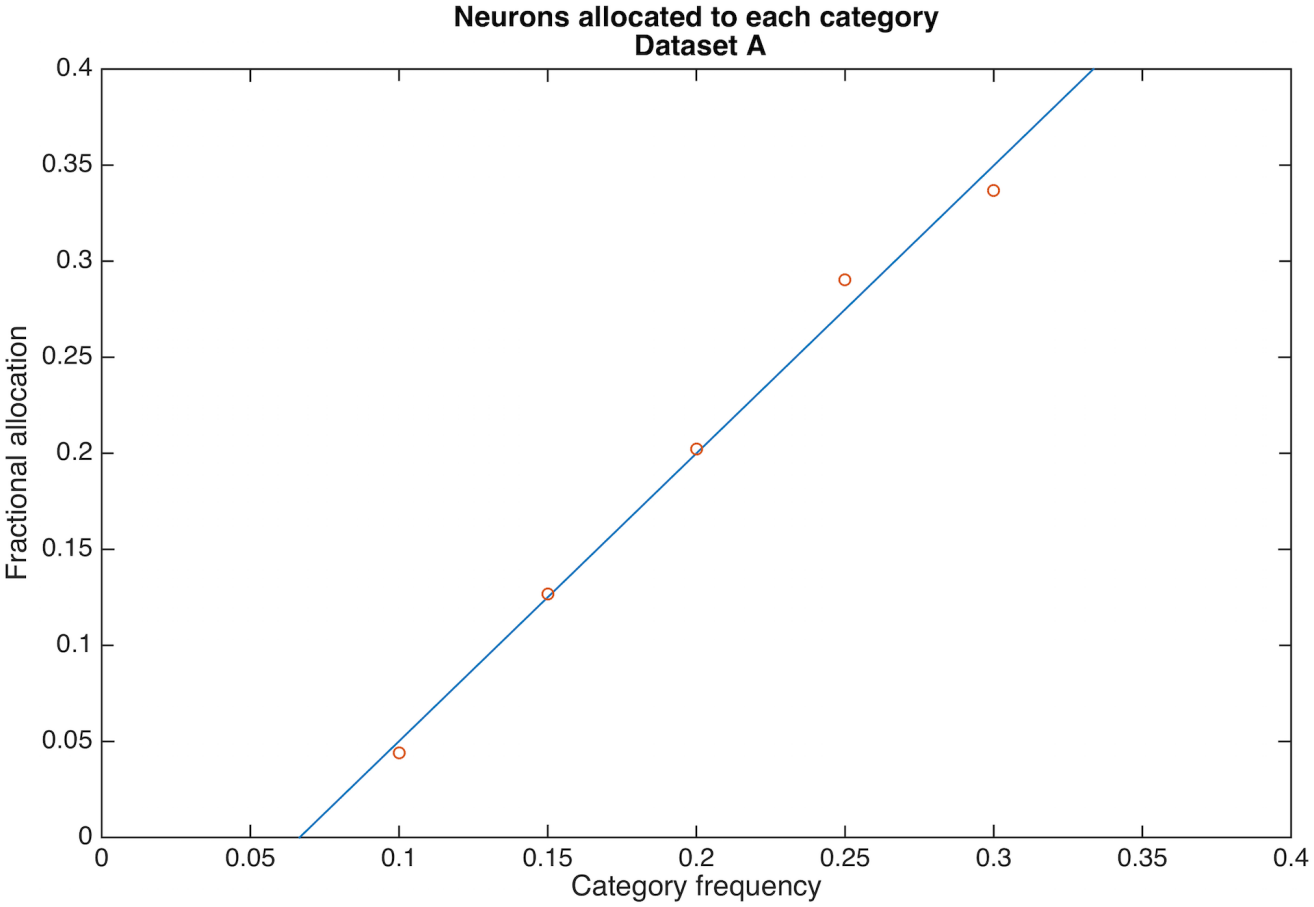
Neuronal allocation is linear in category probability for dataset A. The fraction of postsynaptic neurons firing to a category is proportional to category probability. For this low overlap dataset (see Fig 2A), each postsynaptic neuron fires exclusively to a single category. The linear regression slope is 1.5 (the intercept is -0.1). Each plotted data point is the fraction of 2000 neurons allocated.

The simplest demonstration of the input frequency hypothesis uses dataset A. In dataset A there are five categories, occurring with frequency {.1, .15, .2, .25, .3}. After enough cycles for neurons to stabilize, fractional neuron allocation per category is 0.04, 0.13, 0.20, 0.29, and 0.34, respectively. Thus, higher category frequency does lead to greater neuron allocation, and the relationship is nearly linear as can be seen in Fig 6. With a little thinking, this is an intuitive result for the synaptogenesis and synaptic modification algorithm: Having relatively more inputs belong to a category leads to more postsynaptic neurons learning that category because there is a competition ongoing, a computation biased by category frequency (i.e. higher activity inputs tend to chase-off lower activity inputs through the associative modification equation and shedding).

Although this allocation result clearly supports the basic contention of this paper, the simplicity of this observation belies an additional complexity that occurs when categories are not orthogonal in the input domain.

**Fig 7:**
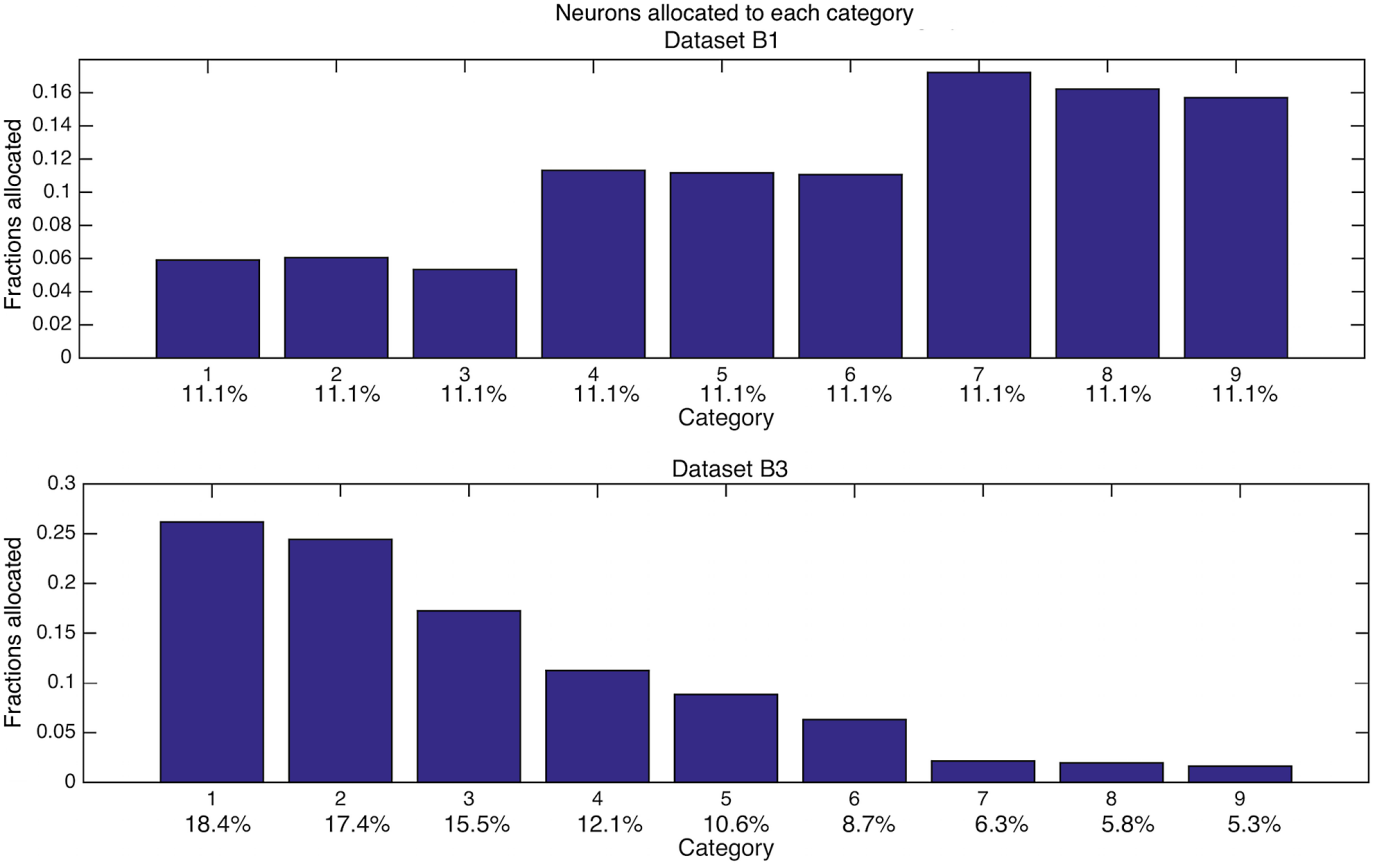
Category frequency can overcome the advantage of highly overlapping super-categories in capturing post-synaptic neurons. (A) shows neuron allocation for dataset B1. Even though the nine categories are equiprobable, categories capture post-synaptic neurons in a non-equiprobable fashion. The greater the overlap within a super-category, the more neurons that are captured by that super-category's base categories. For each category inside a super-category, there is similar neuron allocation. The x-axis labels both category and category frequency (e.g. 11.1%, 11.1%, 11.1%…). (B) shows neuron allocation for dataset B3. By changing the category frequencies, the neuron allocations change, and the effect of overlap is overcome. The change in frequency leads to more neurons allocated to categories with the highest frequency, even inside a super-category. The x-axis labels both category and category frequency (e.g. 18.4%, 17.4%, 15.5%…). Numbers 1 through 9 on each x-axis correspond, in sequence, to the three base patterns comprising the three, sequential super-catergories.

As the datasets become more complicated, category frequency alone will no longer have a simple linear relationship with neuron allocation, a point made quite strongly by dataset B1. Recall that this dataset consists of nine categories, all with equal frequency, where these nine categories arise from three orthogonal “super-categories” as defined in the methods. For this dataset and its equal probable categories, allocation is quite different across categories, not at all uniform as the equiprobable frequency might have suggested. Fewer neurons are allocated to categories with less overlap, while more neurons are allocated to categories with more overlap (Fig 7A). For dataset B1 the fraction of neurons allocated to the three super-categories is 17.4%, 33.45%, and 49.15%. The fractional neuron allocations for each category (grouped by consecutive super-categories) are {0.06, 0.06, 0.05}, {0.11, 0.11, 0.11}, and {0.17, 0.16, 0.16}. Since the relationships between categories within a super-category are relatively constant (small differences due to the randomization algorithm that built the dataset), there is low allocation variance between categories that share a super-category. From a theoretical perspective, increasing overlap within a super-category increases the largest eigenvalue of the covariance matrix of that super-category, which in turn positively correlates with increased neuron allocation.

#### Interacting category probability with category overlap

Dataset B2 specifically modifies dataset B1 to produce *ca*. equal neuron allocations across all categories. To achieve this equal allocation, the relative frequencies change from equiprobable to 13, 13, 13, 11, 11, 11, 10, 9, and 9 percent. These new frequencies produce corresponding relative neuron allocations of 0.11, 0.12, 0.11, 0.11, 0.11, 0.11, 0.11, 0.11, and 0.11.

Thus, adjusting category frequency compensates for the overlap effect, so that if viewed in isolation, this result appears to contradict the thesis of this paper. Dataset B3 also modifies dataset B1. However, this time the modification produces data that, even in isolation, support the main theme of this paper, i.e. higher frequencies produce greater neuron allocations. To illustrate this point, the category relative frequencies are set to 18, 17, 15, 12, 11, 9, 6, 6, and 5 percent. These frequencies produce relative neuron allocations of 0.26, 0.24, 0.17, 0.11, 0.09, 0.06, 0.022, 0.020, and 0.016, respectively. In this case, neuron allocation is monotonically decreasing across categories as appropriate to the hypothesis (Fig 7B). Thus, even with the more complex input structure of the B datasets, there are category frequency values that qualitatively reproduce the result of the simpler dataset A; i.e., neuron allocation clearly depends on category frequency.

**Fig 8:**
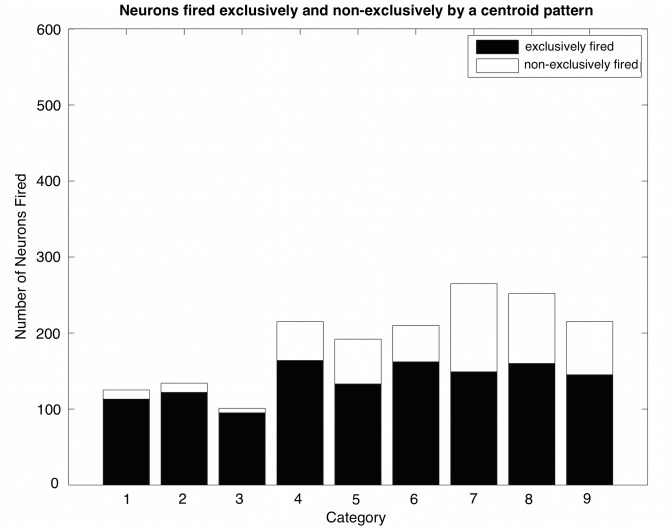
Neurons fired exclusively and non-exclusively by each category’s centroid. All post-synaptic neurons fire exclusively to only one super-category, but some neurons are exclusive to a single category within a super-category (filled bars). Unfilled bars count neurons that fire in response to two or three categories within a super-category. Numbers 1 through 9 on the x-axis correspond, in sequence, to the three base patterns of comprising the three, sequential super-catergories. In all cases, testing threshold is 2.4 because the prototypes are noiseless. Training to B1; testing to full prototype.

For the same simulations using dataset B1, an additional characterization quantifies the fraction of neurons that fire exclusively to each category’s centroid (Fig 8). Note that, regardless of overlap within a super-category, a large number of neurons demonstrate such exclusivity, with values ranging from 95 neurons for the third category to 164 for the fourth category out of 2000 post-synaptic neurons. Unsurprisingly, when comparing such results across super-categories, the fraction of neurons exclusively fired by a category centroid, as a percentage of the total number of neurons fired, decreases with category overlap. For example, the fraction of neurons that fire exclusively to the one of the three category centroids of super-category I is greater than the fraction of neurons that fire exclusively to one of the three categories of super-category III. Even so, regardless of super-category, there are more than enough exclusive neurons for a supervised decoder to capture the information needed for selective recognition across all nine categories.

**Fig 9:**
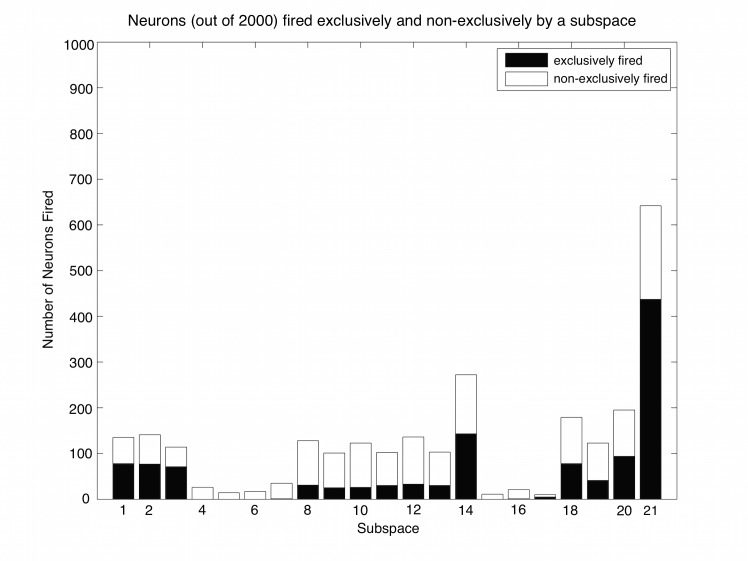
Neurons fired exclusively and non-exclusively by each sub-region. The sub-regions, as in Fig 3, can be learned in an exclusive fashion. Such novel learning is poorest for super-category I; Exclusively fired neurons do not exist in sub-regions I-4, I-5, and I-6. Nearly all of the exclusive neuron firing in super-category I occurs in sub-regions I-1, I-2, and I-3. Super-category II has an even distribution of neurons firing exclusively to II-1 through II-6, while II-7 garners approximately twice the number of neurons as any of the other sub-regions. A majority of neurons that learn super-category III are fired by sub-region III-7 (the triple overlap) and the majority of these neurons fire exclusively. There are a sizeable number of neurons that are learning to fire to III-4, III-5, and III-6 exclusively. Such results are dependent on synapse number per neuron. The 21 sub-regions arising from the totality of all super-categories are numbered here as in Fig 3 but expanded by the sequence of the three super-categories.

A third and more subtle characterization of the neuronal allocation quantifies the neurons that respond to the sub-regions of input space created by the overlap between the nine categories (see Fig 3). The number of neurons fired by sub-regions, exclusively and non-exclusively, is presented in Fig 9. Neurons that fire in response to individual super-category I sub-regions do so almost entirely in a non-exclusive manner. In super-category III, more than one quarter of the neurons firing to sub-region II-7 (the triple overlap) are firing exclusively and zero neurons are firing exclusively to sub-regions III-1, III-2, and III-3. On the other hand, the most reliable exclusive recognition of the sub-regions occurs for super-category II. In super-category II (sub-regions II-1 through II-6), the vast majority of firings are non-exclusive, but there are also many neurons firing exclusively to each sub-region. Across sub-regions II-1 through II-6, the exclusively fired neurons are nearly uniformly distributed.

In sum, the higher the overlap inside a super-category, the more likely there will be neurons learning the highly overlapped sub-region and the less likely there will be neurons learning the non-overlapping sub-regions. But as is the case for super-category II, codes can develop that are quite appropriate for learning the sub-regions. Thus, the adaptive algorithm has created codes that refine the environment consistent with the idea that an expert can be self-taught. That is, pattern differences only implicit through relationships within the data will be discriminated by unsupervised neurons.

## Discussion

### Historical perspective

The theory of adaptive synaptogenesis was developed from observations of empirical neuroscience (see [1], [2], and [16] for motivating studies), from the underlying assumption that in order for a neuron to be most useful, its afferent synapses must reflect the statistical structure of the input world, and from one more motivating idea. We assume that there are desirable operating values for balancing costs versus information (e.g. mean firing-rate or mean excitation vs. variance of excitation). Then, as the outcome of the algorithm, adaptive synaptogenesis guarantees such desirable, predetermined values. In this regard, BCM theory led the way, as it explicitly creates postsynaptic neurons with a particular average excitation [5].

In this regard, BCM theory provided the inspiration for adaptive activity control over the long term. More generally, the importance of producing an average activity level in a postsynaptic neuron became clearer with the demonstration [17] that neuron parameters (such a axonal leak current) imply a particular optimum firing-rate in order to maximize the bits per joule of an axon. Given a neuron with such an optimized axon, the values of synaptic excitation must be important in terms of matching dendro-somatic-initial segment computation with the axon’s optimal firing-rate. As well, its synapses should in some sense maximize incoming information [15]. In any event, the BCM algorithm with initial full-connectivity conjoined with an appropriate shedding rule, may well produce identical results to what is found here. Of course spike-timing rules will also work, again assuming full initial connectivity [18].

Indeed, in its earliest version, the utility of adaptive synaptogenesis was understood in the context Barlow’s information-conserving compressive coding idea [19–20], a clearly energy saving transformation with its reduction in both firing-rate and number of neurons while maintaining almost all of the information of the inputs.

The idea of using random connectivity to create network codes has always been part of our synaptogenesis algorithm; in fact, it is the baseline condition in [6–7]. Independently, such ideas have been used to study efficient connectivity distributions [21] and abstract functions [22]. As documented in our early work [7], just random connectivity without shedding is still quite useful for compressive coding. That is, these randomly formed networks produce large values of mutual information while decreasing statistical dependence. However, as documented in the series of articles [6, 8–9], random connectivity with associative modification is inferior to using the algorithm that includes synaptic shedding of small weights.

Although we know of no first-principles theory for optimizing number of synapses, it is clear from synapse count data and the volume penalties incurred by synaptic structures [23] that only a minute fraction of an input space (for example the lateral geniculate as the input to V1) can form synapses with any one postsynaptic neuron in the cerebral cortex. In this light, it may be possible to tune adaptive synaptogenesis to achieve an appropriate range of synapses per neuron.

There are four differences between the adaptive synaptogenesis algorithm used previously and the current version: two of these (A and B below) are improvements that can be applied to the compressive algorithm, a third (C) is a specialization for neurons performing discrimination, and the fourth (D) is largely inconsequential in the context of the data structures used here.

(A) The learned weights are a function of the local covariance matrix [15] instead of the local correlation matrix.
(B) Instead of an asymptotic approach to zero, receptivity goes to zero for finite values of the relevant variable (here, each neuron's firing-rate).
(C) For the discrimination problem, receptivity is controlled by firing-rate, E[*Z*_*j*_], while average internal excitation, E[*Y*_*j*_], controls receptivity for the information-based compression problem.
(D) Finally, the presynaptic synaptogenesis constraint represented by the parameter ‘avidity’ (e.g., see [9]) is largely irrelevant here, and possibly counter-productive, because its purpose is to balance presynaptic territory. If taken too far, such a balance could work against the desired neuronal allocation result. Nevertheless with the incorporation of an appropriate avidity function, it is possible that there will be some small improvement in resource allocation for the problem studied here.

### New results

There are three primary results here: 1) extension of the adaptive synaptogenesis algorithm from data compression to discrimination; 2) documentation of neuronal allocation as a function of a category’s relative frequency and of the statistical input structure; and 3) when suitably formulated, adaptive synaptogenesis produces a stable connectivity in a stable input world.

#### Convergence to stable input connections

As confirmed here for the first time – using the combination of the covariance-based synaptic modification rule and a receptivity rule that gets driven to zero – neuronal allocation can be adaptively controlled by experience in a sensible way. Also for the first time, we show that convergence occurs for connections and their weight values in finite time. Stable connectivity, i.e. convergence in finite time, is required in the context of a hierarchical network like the visual system of the neocortex because code alteration (mappings from inputs to their neural representation) at the bottom of a hierarchy will require that all the subsequent levels of the hierarchy to re-organize their codes. Indeed, some systems have “critical periods”; a critical period turns off the algorithm after a given time interval. Thus, critical periods guarantee stability (even for non-stationary inputs) but do not guarantee activity levels or information rates as is the case for the adaptive synaptogenesis algorithm.

#### Controlling neuronal allocation

The correlations between any subset of input patterns and the frequency of experience of these input patterns have a strong effect on the rate of convergence. In particular, the greater the pattern-overlap within a super-category, the faster the convergence to stable connectivity for neurons coding said super-category (See Table 4). Such a relationship makes sense because with more overlap there is a better chance that a randomly chosen input-line will be retained due to the probability of positive correlation with other input lines. Moreover, fewer synapses will be needed on average to reach threshold. Going in the other direction, relatively isolated patterns are unlikely to win neuronal allocations and in the extreme of low correlation and low probability, neurons acquiring input lines used by an isolated pattern will never converge, and thus, such patterns will receive the smallest neuronal allocation.

The one final issue that might appear as a disconnect between theory and simulations is average firing rates. That is, there is a coarseness of the achieved firing levels compared to the desired levels. In all cases this coarseness can be understood in terms of the probability of the input patterns. For example, suppose that the desired activity level is 10% (i.e. receptivity for new innervation goes to zero once a neuron fires at least 10% of the time). It is quite conceivable (and we have observed such instances) that a neuron could be firing 8 or 9% of the time and then, one more synapse is added and the neuron fires 15% of the time. Such coarseness will be unavoidable when there are relatively few patterns encoded by relatively small number of input lines. Presumably with a higher dimension input system and many more patterns being sampled, there will be more variability, more synapses per neuron, and thus, such large jumps in activity will become less likely. Also contributing to the large excitatory effect of single synapses is the lack of any shunting inhibition and leak effects. Such inhibition will act divisively to downgrade the effectiveness of all activated excitatory synapses, thus biasing development for more synapses per neuron and, in turn, greater precision of actual firing rate compared to desired firing rate.

#### Functional interpretations

The conjecture of energy-efficient brains, constructed from energy-efficient neurons, begs for adaptive processes to control average neuronal activity and to control the information, or the utility, to justify the expense of positive firing-rates and even a neuron’s existence. Studies of compressive coding implicitly, or explicitly, are concerned with optimizing bits per joule. However, when it comes to making decisions, which neurons surely must encode, discriminations are ultimately concerned with some kind of payoff structure generally referred to as utility. Negative error rates can be used as the simplest kind of utility in a discrimination problem. Assuming that all neurons within one class are spread broadly across the input patterns of that class—i.e. in a way such that neurons are equally useful for their cost, the modified adaptive synaptogenesis algorithm can be successfully applied to discrimination problems. Here we picture groups of firing neurons as working together to encode each input pattern, where information inherent in such a group is enough for correct classification. Thus, we do not necessarily require that an individual neuron, by itself, makes a correct classification, although this is certainly very useful. It is also useful when a neuron is one of many that make the classification of the pattern easier by eliminating some of the competing classes. In this regard, the results illustrate (see Figs 8 and 9) the manner in which neuron allocations contribute to various degrees of sub-region specificity.

In general, neural network theories treat discrimination problems as supervised learning problems (e.g. [24]). However, such supervision may not always exist, and – our very point here – it is not always necessary. Again, note in Figs 8 and 9 that some neurons can get classification exactly right even without supervision while other neurons can help eliminate many of the possibilities without supervision. This unsupervised learning of a precise discrimination – and indeed creation of sub-region codes as occurs uniformly for super-category II (Fig 9) –seems relevant to human learning, particularly in the case of becoming an expert where many years of study are required.

To explain the cognitive relevance of such an unsupervised algorithm, we first considered that, in language, the number of terms and therefore the discriminability of some aspect of the world (e.g., Eskimos and snow, Laps and reindeer, see [25]) varies with amount of experience. That is, the more experience one has with some aspect of the world, then the more refined is one’s ability to discriminate among patterns in that aspect of the world. Along with this cognitive idea is the neuroscientific idea: the more neurons devoted to an aspect of the world, the greater a network’s ability to create discriminable neural codes for that aspect. However, by tying language to this cognitive interpretation, the coding problem becomes intertwined with the possibility of supervised learning, including supervised learning without error correction. Thus, rather than an example with supervised learning, a more apt analogy is self-taught discriminations. Consider the chess expert who can discriminate thousands of board positions while only a handful of specific distinguishing names exist [26]. After five to fifteen moves into a named opening, the board positions cease to have names, but the grandmaster can recognize the board position vector as being familiar and as a variant of a particular set of positions previously encountered. For example, after a sequence of 12 unseen moves, the grandmaster can classify the current position as either emerging from the Slav Accepted Alapin Variation or the Catalan opening, but definitely not the Kings Gambit.

Thus, we submit a valid cognitive analogy: the problem studied here is much like a grandmaster chess-player gaining the capability to discriminate unnamed board positions at a glance. The theory of adaptive synaptogenesis predicts that the relative frequency of board positions (amongst games studied) will control the neuronal allocation. But also, board positions that have greater similarity with other board positions (frequencies being equal) will receive a greater neuronal allocation.

## Supporting Information

S1 Dataset Matlab data files illustrated by Fig 2A and 2B. (ZIP)

S1 Code The Matlab program used for the simulations; parameter settings described in the main manuscript are used with this program. (M)
